# The cytotoxic effect of TiF_4_ and NaF on fibroblasts is influenced by the experimental model, fluoride concentration and exposure time

**DOI:** 10.1371/journal.pone.0179471

**Published:** 2017-06-14

**Authors:** Priscila Maria Aranda Salomão, Flávia Amadeu de Oliveira, Paula Danielle Rodrigues, Luana Polioni Al-Ahj, Kellen Cristina da Silva Gasque, Pia Jeggle, Marilia Afonso Rabelo Buzalaf, Rodrigo Cardoso de Oliveira, John Michael Edwardson, Ana Carolina Magalhães

**Affiliations:** 1 Department of Biological Sciences, Bauru School of Dentistry, University of São Paulo, Bauru, São Paulo, Brazil; 2 Department of Pharmacology, University of Cambridge, Cambridge, United Kingdom; Brandeis University, UNITED STATES

## Abstract

**Objective:**

Titanium tetrafluoride (TiF_4_) has shown promising effect in preventing tooth lesions. Therefore, we compared the cytotoxicity of TiF_4_ with sodium fluoride (NaF) (already applied in Dentistry) considering different fluoride concentrations, pH values and experimental models.

**Materials and methods:**

Step 1) NIH/3T3 fibroblasts were exposed to mediums containing NaF or TiF_4_ (from 0.15 to 2.45% F), both at native and adjusted pH, for 6 h. Step 2) NIH/3T3 were exposed to NaF or TiF_4_ varnishes with 0.95, 1.95 or 2.45% F (native pH), for 6, 12 or 24 h. We applied MTT (1^st^ and 2^nd^ steps) and Hoescht/PI stain (2^nd^ step) assays. Step 3) NIH/3T3 were exposed to NaF or TiF_4_ varnish (2.45% F), at native pH, for 6 or 12 h. The cell stiffness was measured by atomic force microscopy (AFM).

**Results:**

Step 1) All cells exposed to NaF or TiF_4_ mediums died, regardless of the F concentration and pH. Step 2) Both varnishes, at 1.90 and 2.45% F, reduced cell viability by similar extents (33–86% at 6 h, 35–93% at 12 h, and 87–98% at 24 h) compared with control, regardless of the type of fluoride. Varnishes with 0.95% F did not differ from control. Step 3) TiF_4_ and NaF reduced cell stiffness to a similar extent, but only TiF_4_ differed from control at 6 h.

**Conclusions:**

Based on the results of the 3 experimental steps, we conclude that TiF_4_ and NaF have similar cytotoxicity. The cytotoxicity was dependent on F concentration and exposure time. This result gives support for testing the effect of TiF_4_ varnish in vivo.

## Introduction

Fluoride has been widely and successfully used for the prevention of dental caries and erosion [1–4]. The major source of fluoride is sodium fluoride (NaF), and its preventive potential at high concentrations is mainly related to the formation of a calcium fluoride-like layer on the tooth [5]. This layer behaves as a physical barrier and acts as a source of fluoride for enamel and dental biofilm during acid challenges. Despite its extensive clinical use, the effect of NaF is sometimes limited. Accordingly, a very intensive fluoridation regime may be required [6,7], especially in cases where the patient has dental erosion or a high risk for caries. Hence, recent studies have focused on fluoride compounds that might have greater efficacy against tooth demineralization, for instance those containing polyvalent metal ions, such as titanium tetrafluoride (TiF_4_).

The potential of TiF_4_ to prevent tooth demineralization has been investigated since 1997 [8]. Its protective effect is related to the formation of an acid-resistant surface coating and an increased fluoride uptake. The coating is rich in hydrated hydrogen titanium phosphate and titanium oxide, which might act as a diffusion barrier [8–11]. The efficacy of TiF_4_ is highly dependent on the low pH of the agent and the type of vehicle. Previous studies have shown that TiF_4_ could significantly reduce enamel erosion at its native pH (pH 1.2), but not at a pH buffered to 2.1 [12] or 3.5 [13]. On the other hand, TiF_4_ varnish (resin-based material for a slow fluoride release) seems to be more efficient than a TiF_4_ solution against tooth demineralization [14,15].

Although there is laboratory evidence about the effectiveness of TiF_4_ in the management of dental erosion [1] and caries [14] compared to NaF, its effect needs to be confirmed clinically. However, the low pH of TiF_4_ might impair its clinical use, because of possible adverse side effects such as an astringent effect on the mucosa. A scanning electron microscopy study has demonstrated that 1% TiF_4_ solution has cytotoxic effects on L929 fibroblasts [16]. However, to our knowledge, no semi-quantitative tests have yet evaluated the effect of TiF_4_ on the viability and morphology of fibroblasts.

Here we aimed to evaluate the toxicity of TiF_4_, in comparison with NaF, on fibroblasts using three different experimental approaches. First, fluoride salts were added directly into the medium, simulating a mouthrinse, and cytotoxicity was assessed by MTT assay. Second, fluoride was applied as a varnish in contact with the medium and cytotoxicity was assessed by MTT and Hoechst 33342/propidium iodide (HO/PI) stain assays. Third, fluoride was applied as a varnish in contact with the medium and cytotoxicity was assessed via measurement of cell stiffness using atomic force microscopy (AFM). In addition, the dependence of the effects of fluoride on pH, fluoride concentration and exposure time was also taken into account.

## Materials and methods

### Cell culture

NIH/3T3 fibroblasts (ATCC^®^ CRL1658™) were cultured in Dulbecco’s modified Eagle’s medium (DMEM; Sigma-Aldrich Co. LLC, St. Louis, USA) supplemented with antibiotics (100 IU ml^-1^ penicillin and 0.1 mg ml^-1^ streptomycin) and 10% (v/v) fetal bovine serum (FBS; GIBCO Laboratories, Life Technologies, Inc., New York, USA), at 37°C in a humidified atmosphere of 5% CO_2_. Enzymatic digestion with 0.25% trypsin (Sigma-Aldrich Co. LLC, St. Louis, USA) was used to harvest cells for experimental analysis.

### Step 1. Effect of fluoride solutions on cell viability

Cells were plated in 96-well microplates at a density of 5 x 10^3^ cells per well (200 μl culture medium per well, six wells per group) and treated with DMEM (containing antibiotics and serum) containing different TiF_4_ or NaF concentrations (at native and adjusted pH values). Table 1 shows the experimental groups. The native pH values were 9.5 and 1.0 for DMEM containing the highest NaF and TiF_4_ concentrations, respectively. The pH of NaF-DMEM and TiF_4_-DMEM solutions was adjusted to 4.5 by addition of 1.28 M phosphoric acid and 0.78 M sodium citrate, respectively [13]. A positive control consisted of medium with no fluoride salt. Cell viability was assayed by MTT assay (see below) after 6 h of treatment (in biological triplicate).

**Table 1:** Step 1. Experimental treatment groups including different fluoride salts, fluoride concentrations and pH.

Fluoride salt	Concentration	pH
NaF	5.42% (2.45% F)	
	2.71% (1.23% F)	9.5 (native pH)
	1.35% (0.61% F)	Or
	0.68% (0.31% F)	4.5
	0.34% (0.15% F)	
TiF_4_	4.00% (2.45% F)	
	2.00% (1.23% F)	1.0 (native pH)
	1.00% (0.61% F)	Or
	0.50% (0.31% F)	4.5
	0.25% (0.15% F)	

### Step 2. Effect of fluoride-containing varnishes on cell viability

Cells were plated in 24-well microplates at a density of 10^4^ (for the MTT assay) and 5 x 10^4^ (for the other assays) per well (1.5 ml culture medium per well) and treated with varnishes containing TiF_4_ or NaF (0.95%, 1.90% and 2.45% F, at native pH) for 6, 12 and 24 h. The varnish was applied to a protrusion (30 mg varnish per well) that was immersed in the medium, allowing fluoride release at a distance of 5 mm from the cells. A positive control consisted of medium with no fluoride. The control medium contained either 1% fetal bovine serum (FBS for the MTT assay) or 10% FBS (for all assays). A negative control consisted of medium containing sodium dodecyl sulfate (only for the MTT assay). The fluoride content of the media was measured by using an ion-specific electrode (Orion Research, Model 9409) and a miniature calomel electrode (Accumet, #13-620-79), both coupled to a potentiometer (Orion Research, Model EA 940), following hexamethyldisiloxane-facilitated diffusion [17,18]. Cell viability was tested by MTT assay or by HO/PI staining, and the cells’ morphology was assessed using hematoxylin and eosin (H+E) staining (in biological triplicate).

### Steps 1 and 2. MTT cytotoxicity assay

The medium above the cells was removed and the wells were washed with phosphate-buffered saline (PBS). Culture medium containing MTT (0.5 mg ml^-1^; Sigma-Aldrich Co. LLC, St. Louis, USA) was added and the cells were incubated with it for 4 h at 37°C in an atmosphere of 5% CO_2_. The formazan crystals produced were dissolved in dimelthyl sulfoxide (DMSO; Synth Labsynth Prods. Ltda, Diadema, Brazil). After 30 min, absorbance at 540 nm was measured using a scanning spectrophotometer (Fluorstar Optima—BMG Labtech, Ortenberg, Germany) [19]. Percentage viability was calculated, considering the positive control as 100%.

### Step 2. Hoechst 33342/ PI (HO/PI) staining

Differential staining with specific fluorochromes was used to distinguish living and dead cells. Cells were trypsinized and suspended in 100 μl PBS and 100 μl of a solution containing 25% PI (1 mg ml^-1^ in water), 50% fluorescein diacetate (1.5 mg ml^-1^ in DMSO), 10% HO (1 mg ml^-1^ in water) (Sigma-Aldrich Co. LLC, St. Louis, USA) and 15% PBS. Ten microliters of the samples were placed in glass slabs and covered with coverslip. The area of analysis was 0.030 mm^2^. Cells were classified as either viable (spherical blue nucleus stained by HO, green cytoplasm stained by fluorescein diacetate, excited at 360 nm), non-viable cells (blue nucleus with apoptotic bodies stained by HO, green cytoplasm) or necrotic (red enlarged nucleus with spherical vesicles stained by PI, excited at 538 nm) using confocal microscopy (63x Leica TCS_SPE). The non-viable and necrotic cells were considered to be dead. The percentages of living and dead cells were quantified [20,21].

### Step 2. Assay of cell morphology

Cells (only after 24h treatment) were fixed with 4% formaldehyde for 10 min and stained with H+E. Cells were examined using an inverted optical microscope (10x, Leica DM IRBE). Cell morphology was classified as follows:

(**-**) No reaction: no evidence of morphological changes.
(+) Mild reaction: some cells with small morphological changes.
(++) Moderate: detachment of some cells, presence of collapsed or rounded cells, few changes in cell number.
(+++) Severe: cellular fragments, severe reduction in cell number, collapsed and rounded cells with surface blebs.
(++++) Very severe: cell lysis, cell contour loss, severe reduction in cell number, few cells preserved, almost all cells collapsed or totally destroyed, most cells shriveled and wrinkled.

### Step 3. Effect of fluoride-containing varnishes on cell stiffness

Cells were plated in Petri dishes (60 x 15 mm), at a density of 3 x 10^5^ (7 ml medium per dish) and treated with varnishes containing TiF_4_ or NaF (2.45% F, at native pH), or placebo varnish, for 6 or 12 h. The varnish was applied to four protrusions attached to the cover of the Petri dish (30 mg per protrusion). The protrusions became immersed in the medium, allowing fluoride release at a distance of 5 mm from the cells. For positive controls, varnish was omitted. Experiments were carried out in biological triplicate (n = 3 dishes for each condition).

After washing with PBS, cells were exposed to HEPES-buffered saline solution (135 mM NaCl, 5 mM KCl, 1 mM MgCl_2_, 1 mM CaCl_2_, 5 mM glucose, 10 mM HEPES, pH 7.4). The stiffness of individual cells (10 curves for each cell) was measured using a Bioscope atomic force microscope (Bruker, Santa Barbara, USA) mounted on Axiovert 135M microscope (Zeiss, Germany) and controlled by a NanoScope IIIa controller. Silicon nitride AFM cantilevers (spring constant 0.01 N/m) with spherical tips (Novascan, Iowa, USA) were used as mechanosensors. A force-distance curve was plotted, relating the bending of the cantilever (or the applied force) to its position relative to the cell. The stiffness of the cell was deduced from the linear slope of this curve (~400 nm). Analyses were carried out using Atomic J Software version 1.6.0 [22], to determine Young’s modulus values.

### All steps. Statistical analysis

The software GraphPad InStat version 2.0 for Windows (Graph Pad Software, San Diego, USA) was used. The MTT, HO/PI and F release data passed the normality test (Kolmogorov-Smirnov test), but not the equality of variances test (Bartlett test, *P* < 0.05). Values for percentage cell viability (MTT and HO/PI) and fluoride release were compared using Kruskal-Wallis test followed by Dunn’s test. Cell stiffness values were compared using ANOVA followed by the post-hoc Bonferroni test. Percentage of viability (MTT) and fluoride release were correlated using Pearson’s test. The level of significance was set at 5%.

## Results

### Step 1

After 6 h, all fluoride treatments reduced cell viability by 100% compared with the positive control (*P* < 0.0001), with no significant differences between the fluoride salts, concentrations, or pH values (Table 2).

**Table 2:** Step 1. Median (interquartile interval) of the % of cellular viability assessed by MTT after treatment with F solutions.

Groups	%		Groups	%	
Control	87.1 (60.8)				
5.42% NaF pH 9.5	0.0*(0.0)		4% TiF_4_ pH 1.0	0.0 (2.6)	
5.42% NaF pH 4.5**	0.0 (10.3)		4% TiF_4_ pH 4.5**	3.6 (8.3)	
2.71% NaF pH 9.5	0.0* (0.0)		2% TiF_4_ pH 1.0	0.0 (1.0)	
2.71% NaF pH 7.5**	0.0 (0.0)		2% TiF_4_ pH 4.5**	0.0 (0.0)	
1.35% NaF pH 9.0	0.0* (0.0)		1% TiF_4_ pH 1.5	0.0 (0.5)	
1.35% NaF pH 8.0**	0.0* (0.0)		1% TiF_4_ pH 4.5**	25.5 (9.3)	
0.68% NaF pH 9.0	0.0* (0.0)		0.5% TiF_4_ pH 3.5	3.6 (7.7)	
0.68% NaF pH 8.5**	0.0* (0.0)		0.5% TiF_4_ pH 5.0**	4.1 (9.8)	
0.34% NaF pH 8.5	0.0* (0.0)		0.25% TiF_4_ pH 5.0	5.2 (13.9)	
0.34% NaF pH 9.0**	0.0* (0.0)		0.25% TiF4 pH 5.5**	0.0 (0.5)	

* shows groups that presented median values statistically different from the median values of the control (Kruskal-Wallis and Dunn tests, P < 0.0001).

** final pH values obtained by using a pH paper indicator, after the serial dilution of the most concentrated fluoride medium.

### Step 2

The fluoride concentrations in the medium as a result of release from the varnishes ranged between 3 and 140 ppm. After 6 and 24 h, the various groups behaved similarly; specifically, TiF_4_ (2.45% F) and NaF (1.90% and 2.45% F) varnishes significantly released more fluoride than the control and placebo. On the other hand, the varnishes with the lowest fluoride concentrations did not differ from the control and placebo (Table 3, *P* < 0.0001). After 12 h, the results among the various groups were similar to those over other periods; however, in this case TiF_4_ (1.90% F) released more fluoride than placebo/control, while NaF (1.90% F) did not. Differences between the experimental times were found for all groups, except for the control, NaF (0.95% F) and placebo.

**Table 3:** Step 2. Median (interquartile interval) of the released fluoride (ppm) from the varnishes to the medium.

Groups	6h	12h	24h
Positive Control	0.01 (0.00)^bA^	0.01 (0.01)^bA^	0.01 (0.01)^cA^
4.00% TiF_4_ (2.45% F)	16.30 (2.18)^aA^	67.11 (55.06)^aB^	31.24 (12.82)^abAB^
3.10% TiF_4_ (1.90% F)	8.12 (5.66)^abA^	53.5 (1.75)^aB^	27.59 (2.18)^abcAB^
1.50% TiF_4_ (0.95% F)	9.25 (1.57)^abA^	15.81 (8.74)^abAB^	19.37 (10.30)^abcB^
5.42% NaF (2.45% F)	16.56 (3.84)^aA^	90.50 (51.47)^aB^	95.79 (54.22)^abB^
4.20% NaF (1.90% F)	17.49 (5.14)^aA^	38.15 (13.70)^abAB^	139.61 (14.50)^aB^
2.10% NaF (0.95% F)	8.52 (5.11)^abA^	2.95 (1.21)^abA^	4.19 (4.77)^bcA^
Placebo	0.01 (0.01)^bA^	0.01 (0.00)^bA^	0.01 (0.02)^cA^

Values in the same column with distinct superscript lowercase letter show significant differences among the treatment groups. Values in the same line with distinct superscript uppercase letters show significant differences among the experimental times (Kruskal-Wallis test followed by Dunn’s test, P < 0.0001).

**Fig 1:**
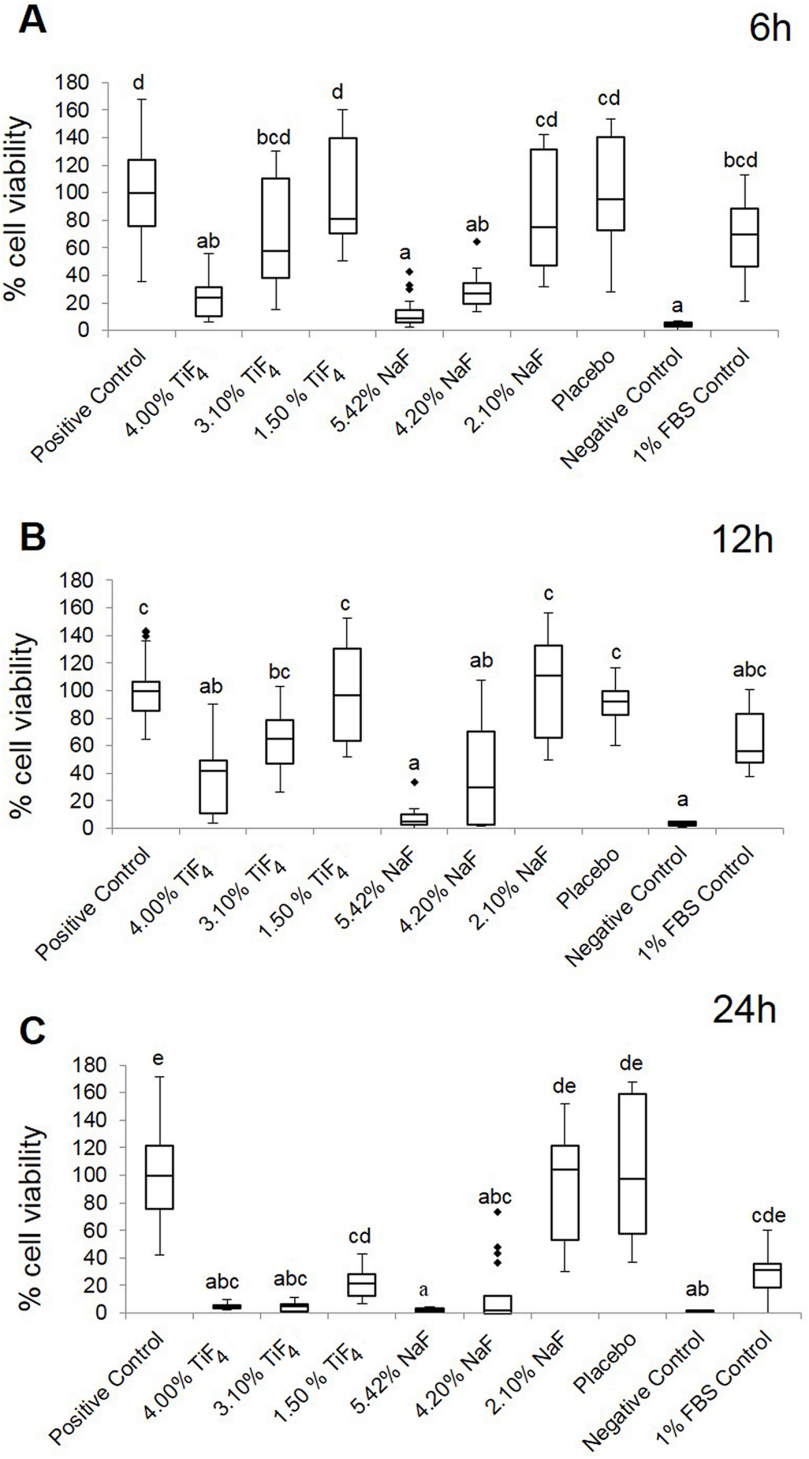
**Step 2. Box plots of the percentage cell viability, according to MTT assay, for the various experimental varnishes’ treatments after (A) 6 h, (B) 12 h and (C) 24 h.** 4.00% TiF_4_ and 5.42% NaF (2.45% F); 3.10% TiF_4_ and 4.20% NaF (1.90% F); 1.50% TiF_4_ and 2.10% NaF (0.95% F). Distinct lower-case letters show significant differences among the treatment groups (n = 6 for each group; Kruskal-Wallis test followed by Dunn’s test, *P* < 0.0001).

As judged by the results of the MTT assay, after 6 and 12 h, TiF_4_ (2.45% F) and NaF (1.90% and 2.45% F) varnishes significantly reduced the percentage cell viability compared to positive control (10% FBS), similarly to the negative control. No significant difference was found between positive control and 1% FBS control. TiF_4_ (0.95% and 1.90% F), NaF (0.95% F) and placebo varnishes were not cytotoxic at these times (Fig 1A and 1B, *P* < 0.0001). After 24 h, the results among the groups were similar to previous periods, but in this case TiF_4_ (1.90% F) varnish did have a cytotoxic effect (Fig 1C, *P* < 0.0001).

No significant differences were found between the fluoride salts, except at 24 h for 1.90% F, in which TiF_4_ was more cytotoxic than NaF. With respect to the experimental periods, the fluoride toxicity was significantly increased after 24 h compared to 6 h and 12 h (except for NaF, 0.95% F); however, toxicities at 6 h and 12 h did not differ from each other.

The percentage cell viability (by MTT assay) and fluoride release from the varnishes showed a moderate but significant inverse correlation (r: -0.66, 95% CI: -0.84 to -0.35, *P* = 0.0004). Hence, cellular viability showed a tendency to decrease as the fluoride concentration of the varnish rose.

**Fig 2:**
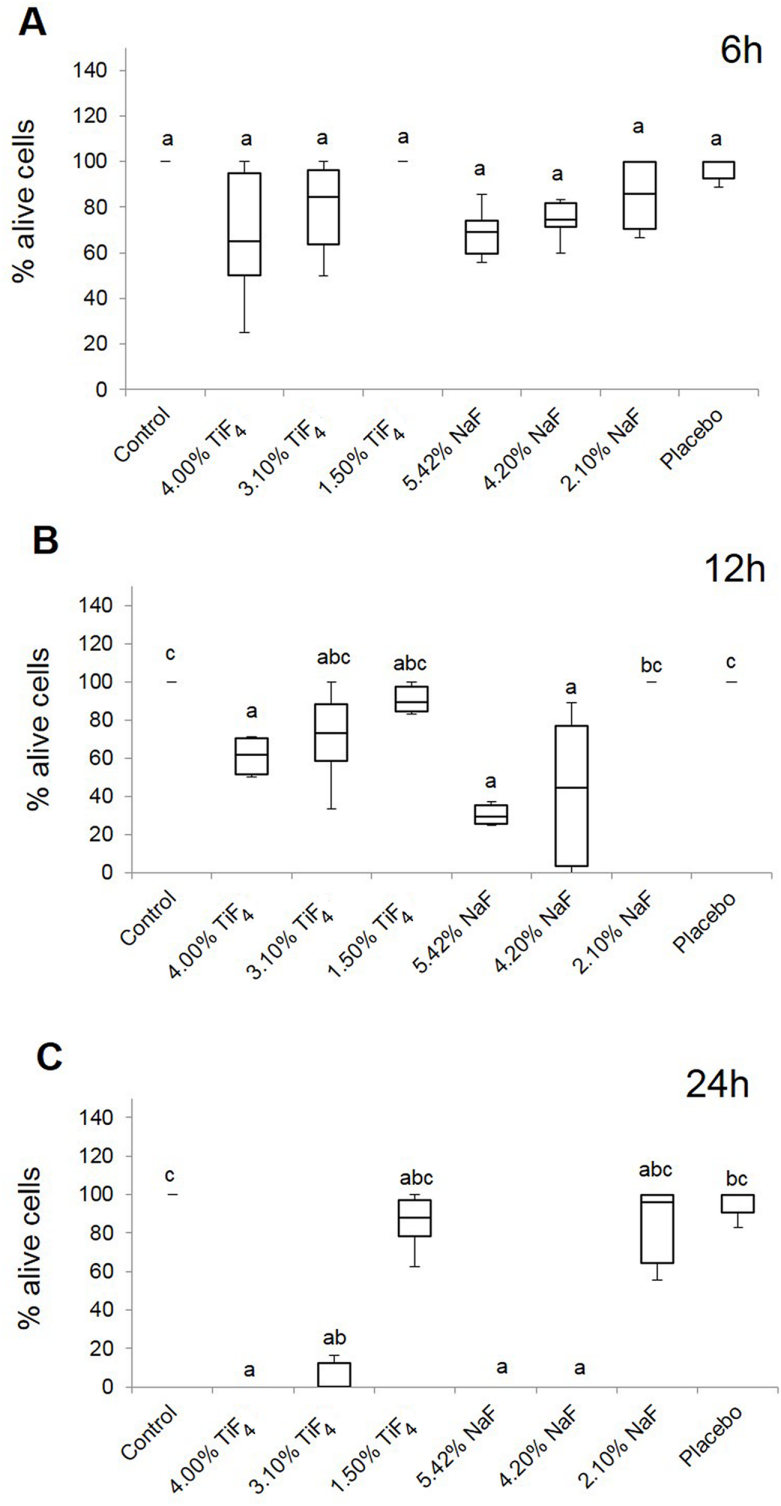
Step 2. **Box plots of the percentage cell viability, according to HO/PI staining, for the various experimental varnishes’ treatments after (A) 6 h, (B) 12 h and (C) 24 h.** 4.00% TiF_4_ and 5.42% NaF (2.45% F); 3.10% TiF_4_ and 4.20% NaF (1.90% F); 1.50% TiF_4_ and 2.10% NaF (0.95% F). Distinct lowercase letters show significant differences among the treatment groups (n = 3 for each group; Kruskal-Wallis test followed by Dunn’s test, *P* < 0.0001). The number of cells found in positive control was: 8.67±1.37 (6h), 9.50 ±2.17 (12h) and 11.83±1.83 (24h).

**Fig 3:**
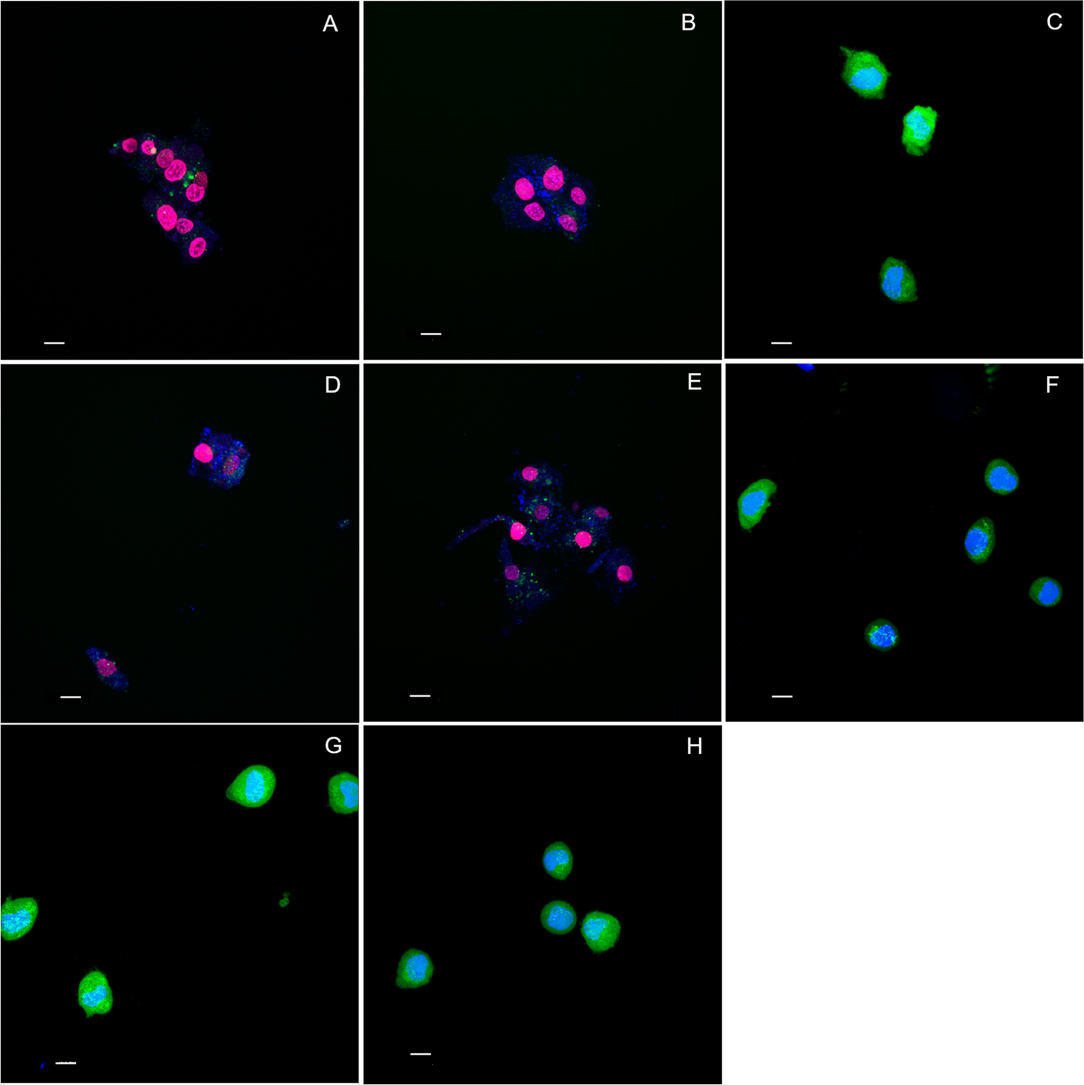
Step 2. **Representative confocal images of the 24 h varnishes’ treatments.** (A) 4.00% TiF_4_, (B) 3.10% TiF_4_, (C) 1.50% TiF_4_, (D) 5.42% NaF, (E) 4.20% NaF, (F) 2.10% NaF, (G) Control, (H) Placebo. 4.00% TiF_4_ and 5.42% NaF (2.45% F); 3.10% TiF_4_ and 4.20% NaF (1.90% F); 1.50% TiF_4_ and 2.10% NaF (0.95% F). Scale bar, 10 μm.

According to the HO/PI assay, after 6 h, there were no significant differences in the percentage of living cells among the various treatments (Fig 2A). After 12 h, the treatments causing a significant reduction in the percentage of living cells were TiF_4_ (2.45% F) and NaF (1.90% and 2.45% F) compared with the positive control (Fig 2B). After 24 h, the results were similar to 12 h, but in this case TiF_4_ (1.90% F) varnish also showed cytotoxicity (Fig 2C). The varnishes with lowest fluoride concentrations (for both TiF_4_ and NaF, 0.95% F) and placebo varnishes were not cytotoxic. Representative images for the 24 h treatments are shown in Fig 3. No significant differences were found between the effects of fluoride salts according to the HO/PI assay. With respect to the experimental periods, fluoride toxicity was significantly increased after 12 h compared with 6 h for NaF (1.90% and 2.45% F), and after 24 h compared with 12 h for TiF_4_ (1.90% and 2.45% F). For the other treatments, there was no significant difference between the periods.

**Fig 4:**
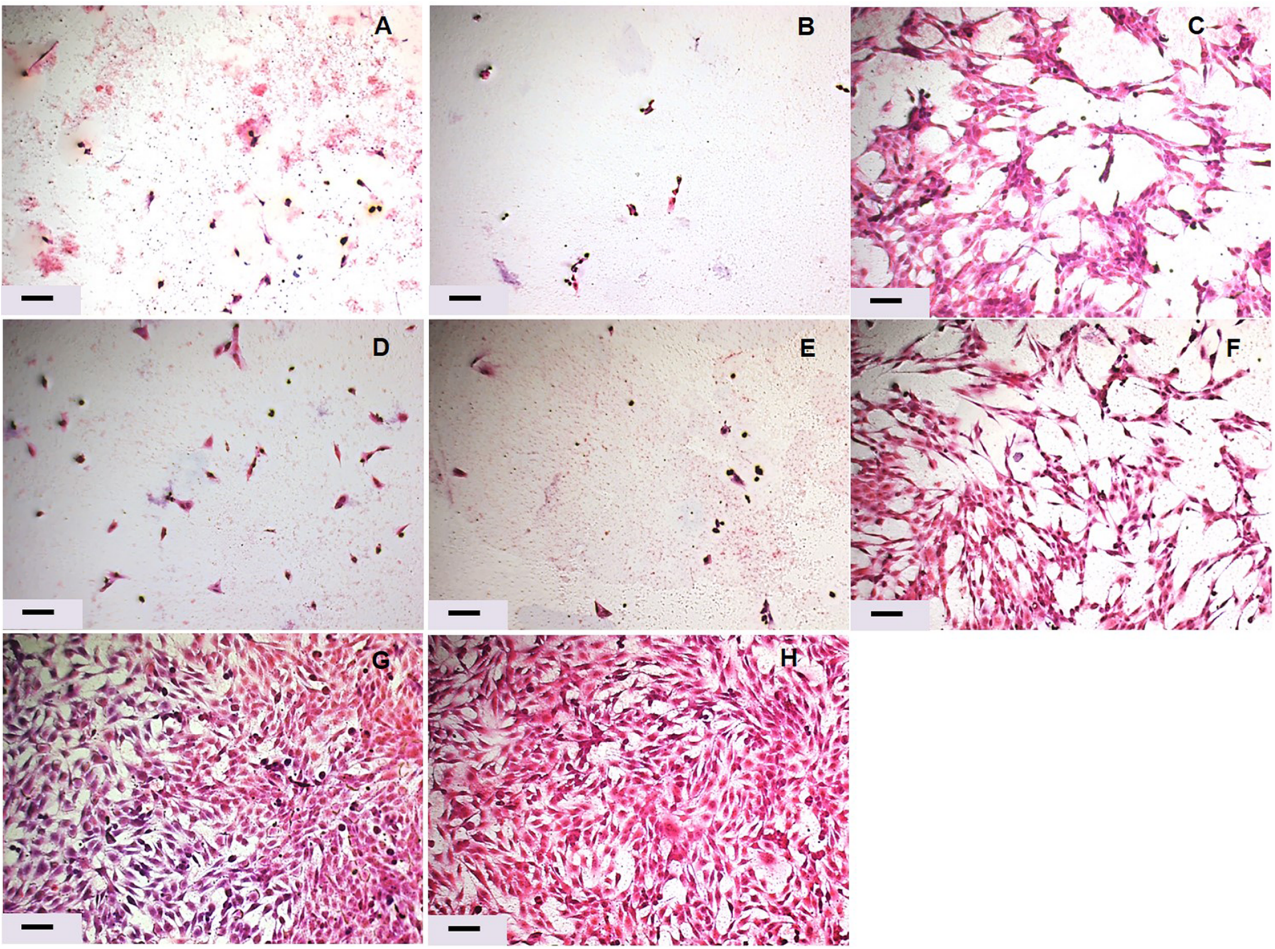
Step 2. **Representative H+E images of the 24 h varnishes’ treatments.** (A) 4.00% TiF_4_, (B) 3.10% TiF_4_, (C) 1.50% TiF_4_, (D) 5.42% NaF, (E) 4.20% NaF, (F) 2.10% NaF, (G) Control, (H) Placebo. 4.00% TiF_4_ and 5.42% NaF (2.45% F); 3.10% TiF_4_ and 4.20% NaF (1.90% F); 1.50% TiF_4_ and 2.10% NaF (0.95% F). Scale bar, 100 μm.

The observed alterations in cell morphology confirmed the results for cell viability. After 24 h, both TiF_4_ and NaF varnishes (1.90% and 2.45% F) drastically reduced the number of cells and caused morphological alterations in the cells that remained, while the lowest concentrated fluoride varnishes (TiF_4_ and NaF, 0.95% F) caused moderate morphological alterations with only slight changes in cell number. No differences were seen between the fluoride salts (Fig 4).

### Step 3

**Fig 5:**
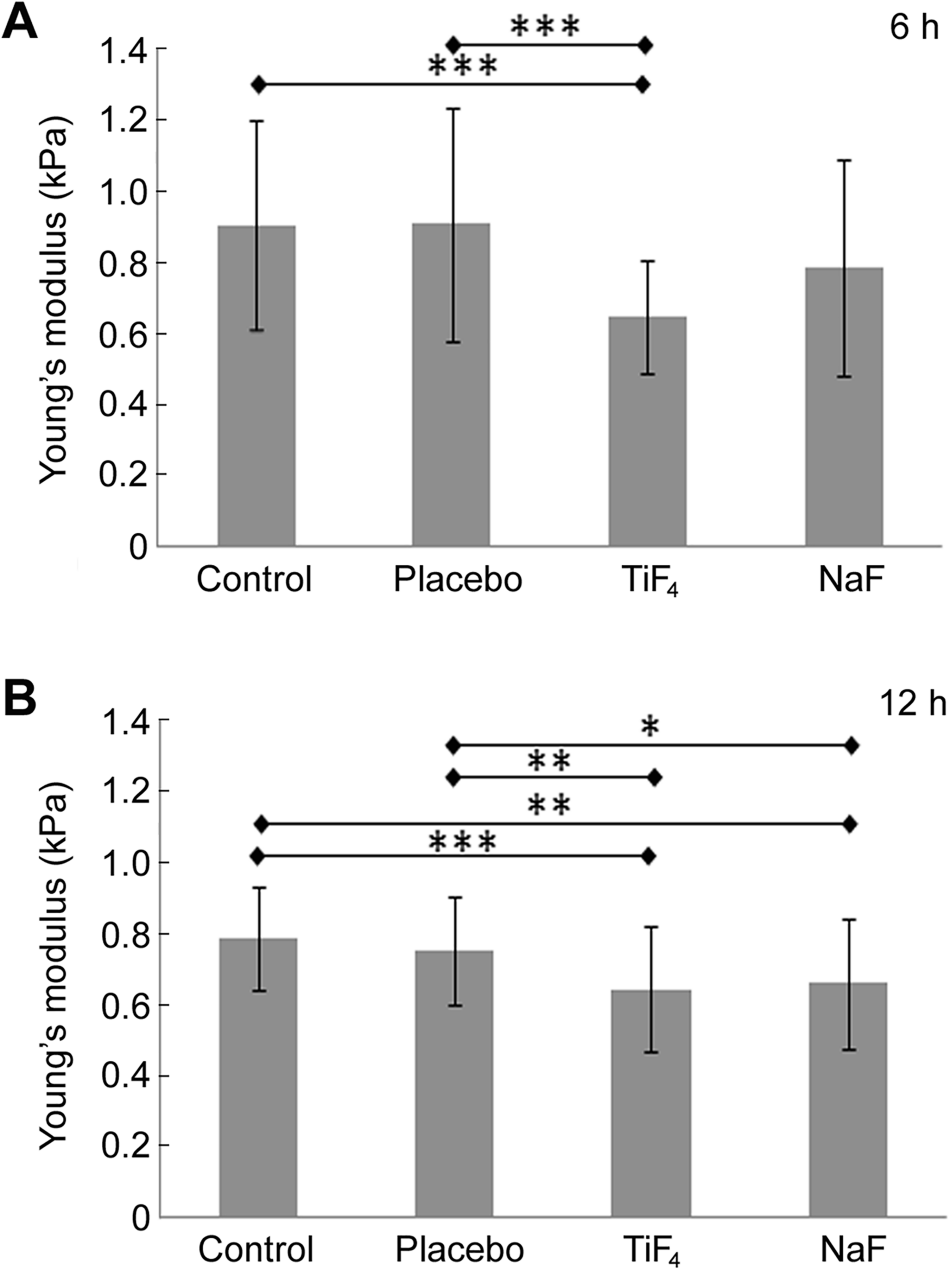
Step 3. **Mean and standard deviation values for cell stiffness (Young’s modulus) for samples subjected to different varnishes’ treatments for either 6 h (A) or 12 h (B).** Asterisks show significant differences between groups (ANOVA, followed by the post-hoc Bonferroni test. * *P* < 0.05; ** *P* < 0.01; *** *P* < 0.001). For (A), Control and 5.42% NaF (2.45% F), n = 45; Placebo, n = 46; 4.00% TiF_4_ (2.45% F), n = 47. For (B), Control, n = 45; Placebo, n = 49; 4.00% TiF_4_ (2.45% F), n = 47; 5.42% (2.45% F) NaF, n = 46.

Over a 6 h period, TiF_4_ varnish (2.45% F) significantly reduced cell stiffness compared with the control and placebo, while the stiffness of cells exposed to NaF varnish (2.45% F) did not differ from the control and placebo. There was no significant difference between the effects of TiF_4_ and NaF varnishes (Fig 5A). Over a 12 h period, TiF_4_ and NaF varnishes significantly reduced cell stiffness compared with the control and placebo (Fig 5B). There was no significant difference between the effects of TiF_4_ and NaF varnishes.

## Discussion

Cytotoxicity assays are important tools in dentistry and should be performed before a novel material comes into clinical use [11,23]. NIH/3T3 has been widely applied in cell culture for several decades, including tests of cell viability and toxicity of biomaterials used in Dentistry as it can be used as a substitute for human gingival fibroblasts [24,25].

TiF_4_ reduces enamel demineralization, and improves remineralization of dental caries lesions [14,15,26,27] and tooth erosion progression *in vitro* and *in situ* [1,12,13,28,29]. It thus seems to be a promising agent for the control of both dental lesions and cytotoxicity studies are warranted to establish its safety. NaF, the most common fluoride compound used in dentistry worldwide, was included in this study to provide a comparison with TiF_4_. It has been shown previously to be cytotoxic depending on the cell type, fluoride concentration, and duration of the treatment [9,30–33].

Despite some studies have shown cytotoxicity of NaF, it has been considered safe especially when included in varnish. Varnish has some advantages compared to mouthrinse or gel, due to its adherence to the tooth surface, allowing a longer contact time between fluoride and enamel (from 6h to 12h). A previous study has shown that NaF and TiF_4_ (2.45% F) varnishes partially release fluoride along to 12h in contact with water and artificial saliva (in a range of 15–25 ppm F) [34], amount of fluoride lower than found in DMEM in this study. Varnish is known to have low toxicity due to the low quantity used during application and to the low absorption in intestine and concentration in plasma. It is also well tolerated and accepted by the patients [35–38].

Our study aimed to compare the cytotoxicity potential of NaF and TiF_4_, both at high and similar fluoride concentrations as those found in products indicated for professional application. Experiments using solutions containing different fluoride concentrations were aimed at simulating the application of TiF_4_ as a mouthrinse. The concentrations were chosen according to previous studies [14,15], which were then reduced (up to 16-fold) to mimic the natural clearance by saliva. We tested solutions at the native pH found in the medium after the addition of the fluoride salt, as well as solutions in which the pH was adjusted to 4.5. Adjustment of pH, as previously reported [13], is important to allow comparison between both fluoride salts under the same experimental conditions, and also for understanding the roles of pH and fluoride concentration in cytotoxicity. Fluoride can cross cell membranes as HF, in response to a pH gradient. Thus, the lower the extracellular pH, the higher the amount of fluoride that enters into the cell [39]. A pH value of 4.5 was chosen since it is the lowest pH value acceptable in fluoride containing products for topical use [40].

It has been reported that the toxicity of TiF_4_ might be related to its low pH [16]. When applied as solutions, TiF_4_ and NaF had comparable cytotoxic effects. Our data disagree with a previous report that 1% TiF_4_ (pH 1.35) at native pH is more toxic than NaF (pH 8.45), which was mainly attributed to the lower pH of the first [16]. In our study, the MTT assay did not show the same trend, which might be due to the fact that all treatments drastically reduced cell viability. In other words, the toxic effect of pH was masked by the presence of high fluoride concentration. It is important to highlight that it is also possible that sodium citrate, used to increase the pH of the TiF_4_ solution, could have a cytotoxic effect similar to those caused by the low pH itself. Thus, it seems that the increase in the pH of TiF_4_ solutions might not benefit the patient, at least not through the use of sodium citrate.

The differences between our TiF_4_ data and previous results [16] could be due to the experimental design. Sen et al. [16] applied fluoride to dentin slices, on which the cells were grown, rather than adding it directly to the medium as we did. The fluoride treatment could have interfered with cell attachment to the slices. Furthermore, Sen et al. [16] used scanning electron microscopy to detect cytotoxic effects. They also used a different cell line, although L929 and NIH/3T3 cells have a similar proliferative rate [41] and viability [42], and so would be expected to respond to fluoride treatment in a similar fashion.

We propose the incorporation of TiF_4_ into a varnish for professional application because varnishes have been shown to be better than an equivalent solution for the prevention of dental caries and erosion [14,43]. In our study, the TiF_4_ and NaF varnishes partially released their fluoride content in DMEM, and we found a negative correlation between fluoride release and viability. In respect to the analyzed variables, the two highest fluoride concentrations tested here were cytotoxic, reducing viability and causing morphological changes, while the lowest fluoride concentration was not. Additionally, a 24 h treatment led to lower cell viability and survival than 6 h and 12 h treatments. On the other hand, TiF_4_ and NaF varnishes were similarly cytotoxic.

Inkielewicz-Stepniak et al. [23] showed a reduction in viability of fibroblasts treated with 1.5 mM but not 0.5 and 1 mM NaF, after 24 h of treatment. Similarly, Tsutsui et al.[33] found that cytotoxicity increased linearly with increasing NaF doses (2.3 to 9.5 mM) and time of exposure (1–24 h). On the other hand, Lee et al. [44] found that doses higher than 20 mM NaF were necessary to reduce cell viability. Our data, considering a range of 0.16 to 7.3 mM F in the medium, are more in accordance with the first two of the above-mentioned three studies.

Vieira et al. [45] have shown that the effect of TiF_4_ against enamel erosion is greater when a highly concentrated solution is applied. Comar et al. [46] also showed that more fluoride is deposited on tooth enamel as the fluoride concentration in TiF_4_ varnishes rises. Generally, dental varnishes contain a range of 2.26% to 6% F [47], and the varnishes remain in contact with the tooth for 6–12 h [47,48]. Hence, the relatively high cytotoxicity that we found at 24 h does not reflect the real-life situation; rather, the 6 and 12 h data support the possibility of testing TiF_4_ varnish in further clinical trials, since it was not shown to be more toxic than NaF varnish, which is already widely used clinically.

Lee et al.[49] and Subbiah et al.[50] have suggested that AFM indentation analysis can be used as a new method for evaluating cytotoxicity. In our study, measurement of cell stiffness by AFM confirmed the similarity between NaF and TiF_4_ toxicities. We analyzed stiffness over a depth of 400 nm, which reveals mechanical alterations related to the cytoskeletal proteins beneath the plasma membrane [51]. Considering that the AFM indentation technique is able to reveal the effects of different treatments on cell elasticity in response to cytoskeleton disruption [52,53], we speculate that the fluoride varnishes could cause partial disorganization of the cytoskeleton, which should be studied in detail further.

The mechanism underlying the cytotoxic effects of fluoride has been extensively investigated. Jeng et al. [11] showed that NaF at concentrations above 4 mM reduces protein synthesis and cytosolic ATP levels, and interferes with mitochondrial function, as also shown in our study by the MTT assay. Lee et al. [44] showed that 20 mM fluoride induced chromatin condensation and DNA fragmentation in human gingival fibroblasts, which would lead to apoptosis. In this previous study, NaF also increased mitochondrial release of cytochrome C into the cytosol, enhanced caspase-9, -8 and -3 activities, increased the cleavage of poly (ADP-ribose) polymerase (PARP), and up-regulated the voltage-dependent anion channel (VDAC). Finally, NaF also up-regulated the Fas-ligand (Fas-L), a ligand of death receptor; Bcl-2, a member of the anti-apoptotic Bcl-2 family, was down-regulated [44], in agreement with a recent study of Inkielewicz-Stepniak et al. [23]. Otsuki et al. [54] demonstrated that NaF enhanced the expression and dephosphorylation of Bcl-2-associated death promoter (Bad) protein. Bad protein forms a complex with carbonic anhydrase II (CAII), and NaF stimulates the detachment of CAII from the Bad-CAII complex and its replacement in a Bad-Bcl-2 complex, inducing apoptosis. Taken together, the studies mentioned above suggest that NaF induces apoptosis through both death receptor- and mitochondria-mediated pathways regulated by the Bcl-2 family. The cytotoxic mechanism of TiF_4_ is so far unexplored as yet, although it is likely that similar processes are involved.

## Conclusions

Based on the results of the 3 experimental steps, we have shown that TiF_4_ and NaF have similar cytotoxic effects on fibroblast viability, stiffness and morphology. The cytotoxic effects mainly depend on the fluoride concentration and exposure time. This result gives support for testing the effect of TiF_4_ varnish in vivo.
